# Fecal Microbiota and Associated Metabolites Are Minimally Affected by Ten Weeks of Resistance Training in Younger and Older Adults

**DOI:** 10.3390/sports13040098

**Published:** 2025-03-26

**Authors:** Anthony Agyin-Birikorang, Sarah Lennon, Kristen S. Smith, William Van Der Pol, Morgan A. Smith, Casey L. Sexton, Donald A. Lamb, Kaelin C. Young, Christopher Brooks Mobley, Kevin W. Huggins, Michael D. Roberts, Andrew Dandridge Frugé

**Affiliations:** 1 School of Kinesiology, Auburn University, Auburn, AL 36849, USA; 2 Department of Nutritional Sciences, Auburn University, Auburn, AL 36849, USA; 3 Ocean Spray Cranberries, Inc., Middleboro-Lakeville, MA 02347, USA; 4 Biomedical Informatics, UAB Center for Clinical and Translational Science, The University of Alabama at Birmingham, Birmingham, AL 35294, USA; 5 School of Medicine, Stanford University, Palo Alto, CA 94304, USA; 6 Department of Medicine, Division of Gerontology, Geriatrics and Palliative Care, University of Alabama at Birmingham, Birmingham, AL 35294, USA; 7 Department of Physiology, Pacific Northwest University of Health Sciences, Yakima, WA 98901, USA; 8 College of Nursing, Auburn University, Auburn, AL 36849, USA

**Keywords:** short-chain fatty acids, fecal microbiome, resistance training, aging

## Abstract

Preclinical evidence suggests that short chain fatty acids (SCFAs) produced by gut microbiota may impact body composition and muscle growth. While aging is implicated in negative alterations to the gut microbiome, exercise may mitigate these changes. Limited human evidence indicates that resistance training (RT) does not appreciably alter the gut microbiome in older adults, and no human study has examined whether resistance training differentially alters the gut microbiome and associated SCFAs between younger and older individuals. Therefore, we examined whether 10 weeks of RT differentially altered fecal microbiota composition, fecal and circulating SCFAs, and serum markers associated with gastrointestinal integrity in two cohorts of adults. Fecal and serum samples were obtained from untrained younger (22 ± 2 years, n = 12) and older (58 ± 8 years, n = 12) participants prior to and following 10 weeks of supervised twice-weekly full-body RT. Outcome measures immediately before (PRE) and after the intervention (POST) included dual X-ray absorptiometry for body composition, ultrasound for vastus lateralis (VL) thickness, 16S rRNA gene sequencing fecal microbiome data, serum and fecal SCFAs measured by gas chromatography, and serum intestinal fatty acid-binding protein 2 (FABP2), lipopolysaccharide-binding protein (LBP), and leucine-rich alpha-2 glycoprotein (LRG-1) quantified by enzyme-linked immunosorbent assays. Main effects and interactions were measured by repeated measures analysis of variance (group × time; G × T) for all dependent variables, and Spearman correlations were used to explore relationships among changes in relevant outcomes. The intervention significantly increased VL thickness and lean body mass (*p* < 0.05) equally in both groups. Although group differences in microbiome beta diversity were identified, no effects of age, time, or their interaction were observed for the alpha diversity measures. Seven SCFAs were detected in the fecal samples, albeit no significant age, time, or interaction effects were evident. In serum, acetic acid was the only SCFA detected, with no significant age, time, or interaction effects. Serum LRG1 decreased for all participants (*p* = 0.007) with higher levels in younger adults (*p* = 0.015), but no G × T interactions were observed for this marker, serum FABP2, or LBP. No significant correlations were observed among RT-induced changes in muscle mass-related outcomes and changes in fecal microbiome diversity, total or individual SCFAs, or serum FABP2/LBP/LRG-1. These results highlight that 10 weeks of RT largely does not affect fecal microbiota, associated SCFAs, or select markers of gastrointestinal integrity in untrained younger or older adults.

## 1. Introduction

The human gut contains a diverse array of microorganisms, including bacteria, viruses, fungi, and archaea, which inhabit the gastrointestinal tract. The colon hosts the richest microbial community in the human body [1]. Through symbiotic relationships, the gut microbiota contributes to digestion, vitamin synthesis, immune regulation, and protection against pathogens [2]. Environmental factors, including diet, exercise, xenobiotics, stress, and trauma, have been hypothesized to modify the gut microbiome [3,4,5]; interventions may optimize bacteria–host symbiosis to mitigate disease progression [6].

In 2007, Bäckhed et al. [7] used germ-free mice to investigate resistance to diet-induced obesity. It was the first study to suggest the presence of a gut–muscle axis. Others have reported that six weeks of *Lactobacilli plantarum* (*L. plantarum*) supplementation significantly increased grip strength, swimming time to exhaustion, normalized muscle weight, and the percentage of Type I myofibers in mice [8]. Yan et al. [9] subsequently found that the skeletal muscle fiber type was associated with microbiota. Fernandez et al. [10] demonstrated that endurance training increased the number of taxa in 8-week-old mice, which correlated with physical performance. However, neither resistance training (RT) nor endurance training (ET) altered the phyla and family compositions [10]. Cullen et al. [11] reported that RT induced mixed changes in the gut microbiomes of sedentary young adults. These collective results suggest that intestinal microbiota may communicate with skeletal muscle to, in part, regulate the adaptive responses to exercise training.

Acetate, propionate, and butyrate are the most abundant short-chain fatty acids (SCFAs) in humans and are microbial metabolites produced through the fermentation of dietary fibers and resistant starches [12]. Acetate is a bacterial product of the genera *Bifidobacteria*, *Lactobacilli*, *Clostridium*, and *Akkermansia* [13]. Propionate is largely produced by intestinal bacteria of the genera *Bacteroides*, *Ruminococcus*, and *Roseburia* [14]. *Faecalibacterium prausnitzii*, *Eubacterium rectale*, and *Roseburia* spp. produce most colonic butyrate [15]. SCFAs provide an apparent direct link between microbiome-derived metabolites and skeletal muscle physiology [16,17]. In this regard, Lahiri et al. [18] presented evidence of SCFAs regulating skeletal muscle mass and function in germ-free (GF) mice by treating the GF mice in vivo with a cocktail of SCFAs, thus partly reversing skeletal muscle impairment. Interestingly, studies in rodents and humans indicate that ET or combined ET+RT training modulates fecal SCFA content [19,20,21,22]. However, to our knowledge, no study has examined whether RT alone alters fecal and circulating SCFAs in humans.

Although the aforementioned results are promising, there are human data indicating that resistance training may minimally affect gut microbiome outcomes. For instance, our group recently published a study in older individuals indicating that six weeks of resistance training does not affect microbiome diversity or abundances of various taxa [23], and this agrees in principle with other resistance training interventions demonstrating null effects [24,25]. However, our prior results may have been due to either the shorter-term nature of the intervention and/or only examining older individuals who demonstrate a limited capacity to experience changes in the gut microbiome [26].

We previously performed two investigations examining the effects of a peanut protein supplement on training outcomes in younger and older adults [27,28], and both studies indicated that training (but not supplementation) promoted increases in measures of skeletal muscle hypertrophy and strength. Given our interest in determining if longer-term training and/or aging affects gut microbiome adaptations, we leveraged banked fecal specimens and remaining blood sera from 12 younger and 12 older participants in these studies to examine if 10 weeks of RT altered the gut microbiome, fecal and serum SCFAs, and serum markers of gastrointestinal integrity. Our secondary aim was to determine if changes in these outcomes were associated with indices of muscle hypertrophy. We hypothesized that RT would improve alpha diversity in all participants and alter fecal and serum SCFAs in younger, but not older, participants. However, we adopted the null hypothesis that microbiome and SCFA outcomes would not be associated with training outcomes.

## 2. Materials and Methods

### 2.1. Ethical Approval and Participant Eligibility

**Figure 1:**
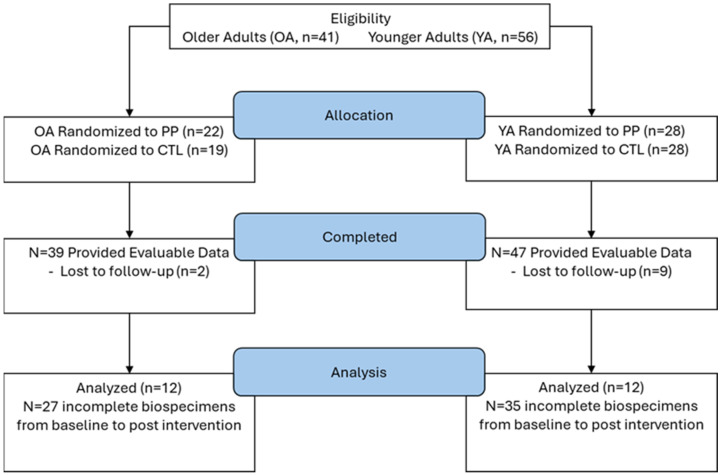
Study flow diagram.

Here, we report a secondary analysis of 12 younger adults and 12 older adults who completed 10 weeks of resistance training. Both studies were conducted under a single approved protocol by the Institutional Review Board (IRB) at Auburn University (AU) (Protocol # 19-249 MR 1907), and both studies were pre-registered clinical trials (NCT04015479 registered 11 July 2019 [28]; NCT04707963 registered 13 January 2021 [27]). All participants signed written informed consent. This sample size was selected from the original 47 younger participants and 17 older participants who finished the 10-week interventions because only these 24 total individuals had enough fecal and blood materials to perform the analyses described in the following paragraphs. See Figure 1.

Detailed methods of the original studies have been previously described [27,28]. Participants were recruited locally and at AU via flyers, emails, and direct contact, either face-to-face or over the phone. Both studies had identical eligibility criteria aside from age, which included (i) being 18–30 years old or 50–80 years old, (ii) body mass index (BMI) < 35 kg/m^2^, (iii) not currently performing RT more than one time per week, (iv) no peanut allergy, (v) no metal implants that could affect X-ray procedures, (vi) minimal radiation exposure in the last six months, (vii) absence of obvious cardiovascular or metabolic disease, (viii) absence of conditions contraindicating participation in exercise program or donation of muscle biopsy, and (ix) exclusion of pregnant females. The eligible persons were informed of all study procedures and completed a medical history questionnaire prior to signing informed consent to participate.

### 2.2. Study Design

This is a brief overview of the procedures relevant to the data presented. Participants reported to AU School of Kinesiology for a pre-testing battery (PRE), which included urine specific gravity (USG) assessment for hydration and a rapid pregnancy test for female participants. Next, weight and height were assessed using digital column scales, right leg vastus lateralis (VL) thickness was measured using ultrasound, and body composition was obtained using a full-body dual-energy X-ray absorptiometry (DXA) scan. Venous blood was obtained from an arm vein in serum separator tubes (BD Vacutainer; Franklin Lakes, NJ, USA) and allowed to clot at room temperature prior to being centrifuged at 3500× *g* for serum separation. The participants were provided 15–30 min education/training on recording dietary intake using a 24 h recall template. They were instructed to provide a three-day log of dietary intake, which was reviewed and verified with study staff at the following visit. They were also provided a stool specimen collection kit with a stool hat, a collection tube with a spoon connected to the lid, and instructions to immediately freeze the specimen after collection. The participants then performed 10 weeks of supervised full-body RT twice weekly, as described by Lamb et al. [28] and Sexton et al. [27]. All training sessions were supervised by the study personnel to ensure proper form and safety throughout the sessions. After completion of the 10-week RT program, the participants were provided with another set of three-day dietary intake forms and stool collection kits for post-intervention testing (POST), occurring 72 h after their last training session. POST replicated PRE procedures, which included USG, a pregnancy test in females, height and weight assessments, right leg VL thickness measurement using ultrasound, a DXA scan, a venous blood draw, and submission of food logs and stool samples. The following paragraphs provide more in-depth descriptions of the testing sessions.

### 2.3. Body Composition

Following an overnight fast, the participants submitted a urine sample for USG level measurement with a handheld refractometer (ATAGO; Bellevue, WA, USA). The participants were provided with water to drink if USG values were less than 1.020. Next, body weight (to the nearest 0.1 kg) and height (to the nearest 0.5 cm) were obtained (Seca 769; Hanover, MD, USA). Following these measurements, a single technician for all measures conducted DXA (Lunar Prodigy; GE Corporation, Fairfield, CT, USA), providing total lean soft tissue mass (LSTM) and fat mass (FM).

### 2.4. Ultrasound for Vastus Lateralis Thickness

Ultrasonography (Real-time B-mode; NextGen LOGIQe R8, GE Healthcare, Chicago, IL, USA) utilizing a multi-frequency linear-array transducer (L4-12T, 4-12 MHz, GE Healthcare, Chicago, IL, USA) captured images of the right mid-thigh vastus lateralis (VL). All ultrasound settings (frequency: 10 MHz, gain: 50 dB, dynamic range: 75), except for depth, were held constant across participants and time points. One image per participant at each time point was obtained. Following the study conclusion, the images were analyzed using the freely available ImageJ software (https://imagej.nih.gov/ij/, National Institutes of Health, Bethesda, MD, USA). VL thickness was measured using the straight-line function and defined as the distance between the subcutaneous adipose tissue–vastus lateralis interface and deep aponeurosis.

### 2.5. Supervised RT Program

The participants completed supervised, progressive RT twice weekly for 10 weeks, where detailed instructions were provided on proper posture, form, technique, and body positioning to ensure safety. The training protocol required a minimum of 48 h in between sessions to allow for appropriate recovery. Each training session was preceded by 10 bodyweight squats and 25 jumping jacks, and warm-up sets as follows: one set of 10 reps at 50% of working weight, one set of 5 reps at 75% of working weight, and one set of 3 reps at 90% of working weight. Younger participants performed either 4 sets of 10 reps (high volume) or 5 sets of 6 reps (high load) at working weight depending on the week. The older participants performed 3 sets of 10–12 reps at working weight. Following each set, the rating of perceived exhaustion (RPE) was recorded to ensure that appropriate loads were implemented throughout. An RPE below 7 resulted in a slight increase in working weight on the next set, and an RPE at 10 or incomplete set resulted in a slight decrease in working weight on the next set.

### 2.6. Dietary Analysis

The participants were instructed to complete three days of dietary intake forms prior to and following the 10-week training intervention, which included all food and beverages on two weekdays and one weekend day. The participants were encouraged to maintain their normal dietary intake. As previously discussed, half of the participants consumed defatted-peanut powder supplements (PPS); the outcomes did not differ between the PPS and control groups. The Nutrition Data System for Research (NDSR; NDSR 2022; University of Minnesota) software was utilized by the study personnel to analyze all young participants’ food logs. The study personnel entered recalls into the Automated Self-Administered 24-h Dietary Assessment (ASA24; National Cancer Institute, Bethesda, MD, USA) tool to analyze all older participants’ food logs.

### 2.7. Fecal Microbiome Analysis

Stool samples were collected using a commode specimen collection kit and sterile collection tubes. The participants were instructed to seal and store samples in the freezer prior to submission at the next visit. Upon receipt, the samples were stored at −80 °C prior to processing. Fecal microbial DNA was isolated using commercially available kits (Zymo, Irvine, CA, USA). DNA samples were prepared, and polymerase chain reaction (PCR) amplified 250 base pair variable region 4 of 16S rRNA. The PCR amplicon library was sequenced on Miseq (Illumina, San Diego, CA, USA) [29,30]. Further detailed processing steps have been previously described [23]. The Quantitative Insight into Microbial Ecology (QIIME) 2 pipeline [31] grouped the demultiplexed FASTQ files and UCLUST clustered sequences into PyNAST-generated Amplicon Sequence Variants (ASVs) with DADA2, ensuring a 97% similarity threshold [32,33,34]. Thereafter, taxonomic assignments were issued using the SILVA database [35]. ASVs with an average abundance >0.005% were processed and grouped by taxonomy.

### 2.8. Fecal and Serum SCFA Analysis

Fecal and serum samples were shipped on dry ice to a commercial vendor (Microbiome Insights, Richmond, BC, Canada) for SCFA analysis. Briefly, SCFAs were quantified using gas chromatography (Thermo Trace 1310) coupled with a flame ionization detector (Thermo Fisher Scientific, Waltham, MA, USA), as previously described [36]. Aliquots of stool samples were homogenized using MP Bio FastPrep post-suspension in MilliQ-grade water at 4.0 m/s for 1 min. Next, the samples were acidified by adding 5M HCl to the fecal suspensions until they reached a final pH of 2.0. The suspensions were incubated for 10 min and then centrifuged at 10,000 rpm. Supernatants were spiked with 2-ethylbturyic acid until a final concentration of 1 mM was reached. SCFAs were detected from the supernatants by direct injection into the Thermo TG-WAXMS A GC Column (30 m, 0.32 mm, 0.25 μm). Standard solutions of individual SCFAs were injected and used for calibration. Serum SCFAs were also analyzed using a similar process, as previously described [37].

### 2.9. Serum Biomarkers

The serum samples were stored and removed from −80 °C to determine concentrations of intestinal fatty acid-binding protein (FABP), leucine-rich alpha-2-glycoprotein 1 (LRG-1), and lipopolysaccharide-binding protein (LBP) using enzyme-linked immunosorbent assay (ELISA) kits according to the manufacturer’s instructions (AFG Bioscience, Wood Dale, IL USA; catalog #’s: EK710951, EK710885, EK241481). All three assays followed similar protocols, which ended with determining absorbance readings at 450 nm using a microplate spectrophotometer (Biotek Synergy H1 hybrid reader; Agilent, Santa Clara, CA, USA). Sample absorbance readings were compared to standard curve absorbance readings, and the values were provided in serum concentration units. The three kits performed adequately, as determined by unknown absorbance values falling within the linear range of the standard curves and coefficient of variation values for duplicates being less than 25% (FABP = 22.8%, LBP = 12.3%, LRG-1 = 18.5%).

### 2.10. Statistics

Statistical analysis was conducted on non-microbiome data using Graphpad Prism v10.0, and microbiome data were analyzed using RStudio Team (2023) (Integrated Development for R. RStudio v2023.06, PBC, Boston, MA, USA). Repeated measures two-way (group × time; G × T) analysis of variance (ANOVA) tests were performed for all longitudinal measures. For microbiome data, false discovery rate (FDR)-corrected Kruskal–Wallis ANOVAs compared the relative abundance of all ASVs between both groups at both time points. Alpha diversity was measured using ASV counts, PD whole tree phylogeny, Simpson index, and Shannon index. Beta diversity was measured using the Bray–Curtis dissimilarity, unweighted Unifrac distance, and weighted Unifrac distance metrics. Change scores for select dependent variables were calculated by subtracting PRE values from POST values. Spearman correlations were then conducted on select change scores to explore the relationships between changes in muscle hypertrophy outcomes versus changes in dietary nutrients, fecal microbiome diversity, fecal SCFAs, and serum markers. Correlations were performed for each age cohort individually, as well as for all participants. Statistical significance for two-way ANOVAs was established as *p* < 0.05, and significance was adjusted to 0.05/576 = *p* < 0.000087 for correlation analyses to account for the high number of associations.

## 3. Results

### 3.1. Participant Characteristics and RT Outcomes

Table 1 presents the baseline characteristics and training adaptations in both the younger and older groups. Significant main effects of time, but no significant interactions, were evident for DXA LSTM and VL thickness, indicating that training increased muscle mass regardless of age group. There were no significant main effects or G × T interactions for total calories, carbohydrate, and fat intake. However, protein and fiber intake increased in both the older and young participants.

### 3.2. Fecal Microbiota Between Age Groups with Resistance Training

**Figure 2:**
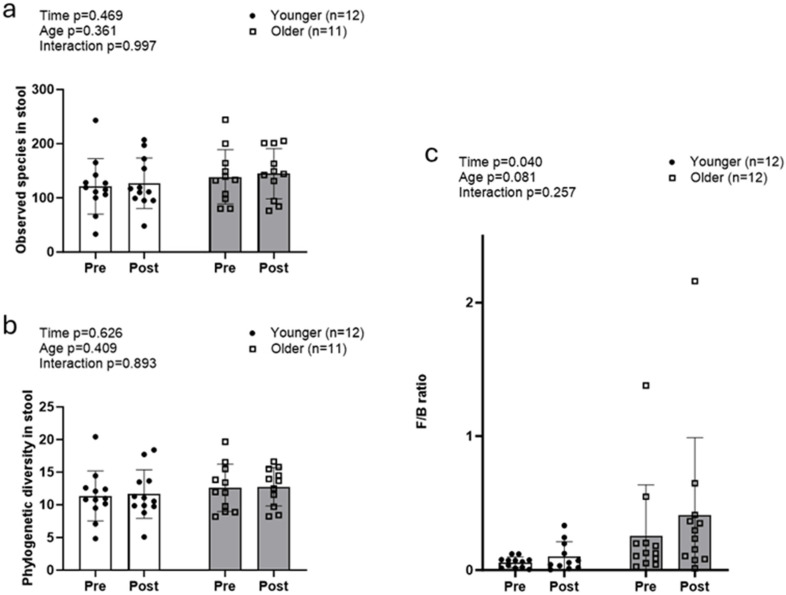
Changes in fecal microbiome alpha diversity with resistance training. Data in this figure indicate that there were no significant changes observed with fecal alpha diversity, including observed species (**a**) and whole tree phylogeny (**b**) in both older and younger participants. However, the Firmicutes/Bacteroidetes ratio (**c**) significantly increased in both groups.

Alpha diversity metrics (observed species and whole tree phylogeny) did not exhibit significant main or interaction effects (Figure 2a,b). The Firmicutes/Bacteroidetes (F/B) ratio increased, exhibiting a significant main effect of time (POST > PRE, *p* = 0.040), but no significant age or interaction effects (Figure 2c).

### 3.3. Beta Diversity Between Age Groups with Resistance Training

**Figure 3:**
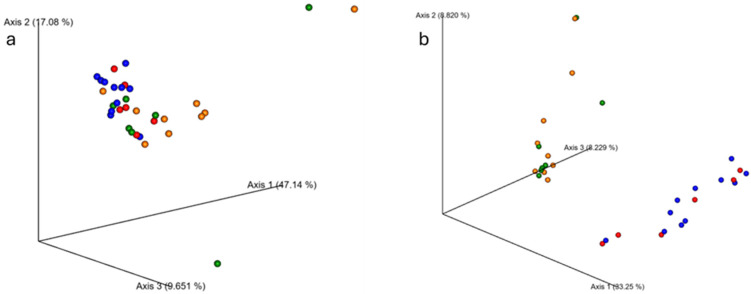
Changes in fecal microbiome beta diversity with resistance training. The average microbiome of young participants was significantly different from the older participants according to (**a**) Bray–Curtis (*p* < 0.001) and (**b**) weighted Unifrac (*p* < 0.001) metrics. Ten weeks of RT did not alter the microbiome composition in either group. Older PRE: red dots; older POST: blue dots; younger PRE: orange dots; younger POST: green.

Though alpha diversity did not differ between groups, younger and older samples did exhibit microbial population differences between the groups (Figure 3). Beta diversity analyses using Bray–Curtis and weighted Unifrac metrics indicated significant differences between younger and older groups (*p* < 0.001 for both), but no differences in composition from PRE to POST (*p* > 0.05 for all).

### 3.4. Serum and Fecal SCFAs Between Age Groups with Resistance Training

**Figure 4:**
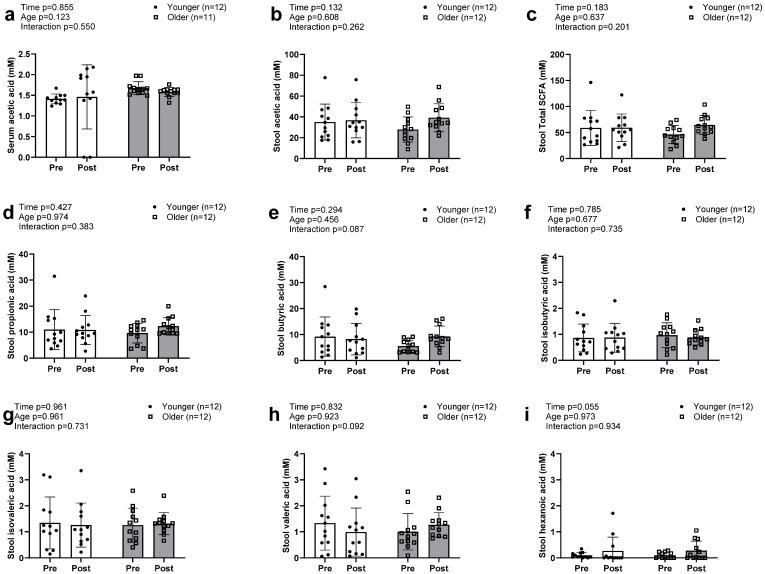
Changes in serum and stool short chain fatty acids with resistance training. Legend: Data in this figure indicates that only acetic acid (**a**) was found in the serum sample and there was no alteration observed between groups. Total SCFAs in the stool sample (**b**) were also not altered between groups. No changes in individual stool SCFAs, including acetic (**c**), propionic (**d**), butyric (**e**), isobutyric (**f**), isovaleric (**g**), and valeric acid (**h**), were observed. However, there was a trend towards a significant increase in hexanoic acids (**i**) in both groups.

Acetic acid was the only SCFA detected in serum in both older and younger participants, with no significant main effects or G × T interaction (Figure 4a). Seven SCFAs were detected in stool samples and included the three highly enriched acetic acid (Figure 4c), propionic acid (Figure 4d), and butyric acid (Figure 4e), as well as the less enriched isobutyric, isovaleric, valeric, and hexanoic acids. No significant main effects or G × T interactions were observed for the seven individual fecal SCFAs or total stool SCFA concentrations (Figure 4b).

### 3.5. Serum Biomarkers of Intestinal Barrier Function

**Figure 5:**
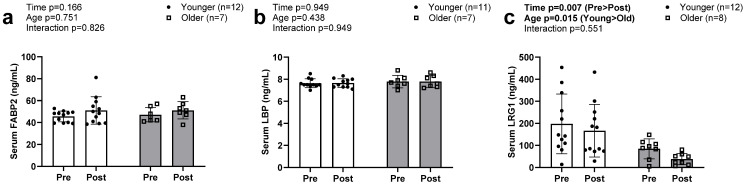
Serum FABP2, LBP, and LRG1 at PRE and POST in older and younger participants. Data in this figure indicate that serum FABP2 (**a**) and serum LBP (**b**) were not significantly altered in either older or younger participants. However, serum LRG1 (**c**) significantly decreased in both groups. Abbreviations: FABP2, fatty acid binding protein; LBP, lipopolysaccharide binding protein; LRG1, leucine-rich alpha-2-glycoprotein 1.

No main effects or interactions were evident for FABP2 or LBP (Figure 5a,b). Serum LRG levels decreased from PRE to POST intervention in both groups (Figure 5c, *p* = 0.007). There was also an observed main effect of group, with older participants exhibiting lower values compared to younger individuals (*p* = 0.015); however, there was no G × T interaction (*p* = 0.551).

### 3.6. Correlations Among Dietary Intake, Fecal/Serum Measures, and Resistance Training Adaptations

**Figure 6:**
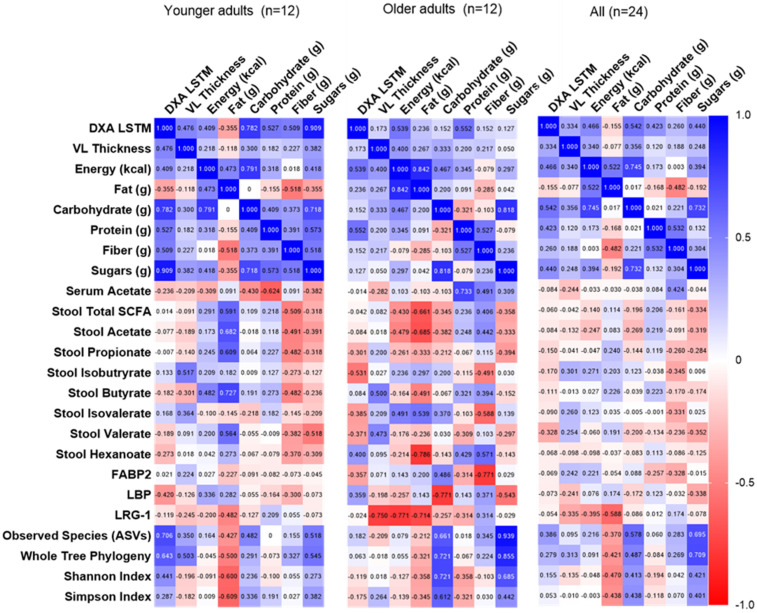
Correlations among changes in primary and secondary outcomes. Data are Spearman rho values. No correlations were significant after adjusting for multiple comparisons; 0.05/576 = *p* < 0.000087. Abbreviations: DXA LSTM, lean/soft tissue mass determined using dual-energy X-ray absorptiometry; obs. species, observed species; SCFA, short chain fatty acids.

Spearman correlations were performed among POST-PRE change scores for key dependent variables in the younger, older, and all participants (i.e., diet composition, fecal/serum SCFAs, fecal microbiome, and serum biomarkers; Figure 6). After adjusting for multiple comparisons, no correlations were statistically significant. However, the following exploratory findings are noteworthy: Lean muscle mass alterations were associated with changes in alpha diversity, and a stronger relationship was observed in younger compared to older adults. Dietary fat in younger adults and dietary sugar in older adults had the strongest associations with alpha diversity. Surprisingly, no relationships were observed between dietary or lean mass changes and serum and stool SCFAs. Serum biomarkers were most strongly associated with measures in older but not younger adults, with inverse relationships observed between LRG-1 and VL thickness, total dietary calories, and fat. Dietary carbohydrate, but not fiber, was inversely associated with LBP, whereas fiber was inversely associated with FABP2 (in older but not younger adults).

## 4. Discussion

This is the first known study to determine the effects of 10 weeks of RT on fecal microbiome, serum and fecal SCFAs, and gut-associated biomarkers in younger and older untrained adults. We observed an increase in LBM and VL thickness in both groups after the 10 weeks, which validated the effectiveness of the training program. The younger cohort presented a greater microbiome diversity than older individuals which did not change with RT. While many training studies have utilized ET interventions in younger adults, there are several studies that have now employed older participants and/or incorporated RT interventions. Taniguchi et al. [38] performed a five-week ET intervention in healthy elderly men and reported that the training program did not change alpha or beta diversity. Alternatively, Bycura et al. [25] examined the effects of the gut microbiome using both 8 weeks of ET and RT interventions in healthy college-aged adults. They found that changes in microbiota differed between training interventions. Although no significant changes in gut microbiota were observed overall, beta diversity analyses revealed notable shifts during the first few weeks of ET. However, post-intervention, these changes slightly regressed, with the microbiota composition resembling the baseline more closely. Similarly, a 6-week ET intervention in obese and lean young adults conducted by Allen et al. [22] supports the notion that beta-diversity changes are dependent on BMI. Additionally, following the 6-week sedentary washout period, the authors reported that changes in the microbiota had reverted to PRE-intervention levels.

Cullen at al. [11] conducted a 6-week resistance training intervention in healthy young sedentary adults and found that RT elicited mixed changes in alpha and beta diversity measures. Our data and previous data utilizing RT interventions collectively suggest that microbiome diversity is not appreciably altered with RT. Nonetheless, the reasons why ET greatly impacts microbiota composition compared to RT should be further investigated.

In our study, RT produced no significant changes in the seven detectable fecal SCFAs and one detectable serum SCFA. These findings are contrary to preclinical rodent studies suggesting the potential involvement of SCFAs in mitigating exercise adaptations. For instance, Huang et al. [39] treated mice with acetate, propionate, and butyrate for four weeks to investigate endurance capacity. They also reported that a higher concentration of SCFAs improved average running time compared to the control. Okamoto et al. [40] also investigated the effects of SCFAs on endurance training outcomes. The authors administered either a low or high microbiome-accessible carbohydrate (LMC or HMC) diet to 10-week-old mice for 6 weeks. They found that the LMC diet group had lower SCFA concentrations and slower treadmill running times compared to the HMC diet group.

The differences in diet composition and SCFA production are consistent with the study by Eveleens Maarse and colleagues [41], who supplemented older adults with a fiber-rich dietary mixture of acacia gum and carrot powder (30 g/day) and observed an increase in fecal SCFAs in the intervention group versus placebo. Estaki et al. investigated the effects of SCFAs on aerobic capacity in humans. They concluded that healthy young adults with higher SCFA concentrations had a higher VO_2_ peak [42]. These studies suggest that changes in SCFA correlate with changes in exercise performance. However, our training involved resistance exercises rather than endurance exercises, which could explain the differences in the previously discussed results.

Given our investigation of the gut microbiome and SCFAs with RT interventions, it was ideal to assess biomarkers of intestinal barrier integrity. Although serum LBP was unchanged, we observed a trend toward increased serum FABP2 following RT. Numao et al. [43] reported similar findings, as they also observed an increase in FABP after low- and moderate-intensity exercise in healthy men. However, the authors utilized an aerobic training intervention. Additionally, we observed a significant decrease in LRG1 levels with RT intervention. Elevated levels of LRG1 in the bloodstream are often associated with inflammatory diseases, cancer, and cardiovascular disorders, making LRG1 a potential biomarker for disease activity [44,45,46]. Our findings suggest that RT could improve intestinal barrier integrity, and this should be further explored.

### Limitations

Though this study provides new evidence for the gut–muscle axis in humans, it does have limitations. First, our sample was fairly homogeneous, as there were 63% white participants and 75% female participants. It would be valuable to investigate whether race or gender is a factor in gut microbiota composition and whether SCFAs change with training. Another limitation is that the older cohort had significantly greater total body mass, which is typically associated with dysbiosis [47]. While our sample included participants in an effective RT program, there was no control group that did not train, and our sample (n = 24) was relatively small. Importantly, the relative training volume was not equal for the two cohorts compared. We also did not request that participants collect stool samples at the same time of day, which may have affected fecal microbiome composition [48]. Another important limitation of our study was the lack of dietary guidance; the variance in fiber alone constitutes a major confounding factor that could have influenced these results. Future investigations should include personalized dietary interventions that allow standardization of dietary intakes, perhaps during a run-in period prior to RT. Finally, while we educated participants on completing accurate diet logs, self-reporting was a major limitation for our diet analysis [49].

## 5. Conclusions

This is the first known study to investigate the effects of RT on the fecal microbiome and SCFAs in humans. Alpha diversity and beta diversity analyses indicate that 10 weeks of RT did not change the overall microbiome composition of untrained adults, though younger and older participant cohorts had differing compositions across time. Fecal and serum SCFAs were not significantly altered after 10 weeks of the RT program. Additionally, fecal SCFAs did not translate to serum SCFAs, potentially due to utilization by colonocytes or maintenance of intestinal epithelium integrity. Although these results are preliminary, we posit that RT adaptations in humans are likely mitigated by factors other than the fecal microbiome or its metabolites.

## Figures and Tables

**Table 1:** Participant characteristics, training outcomes, and self-reported food intakes.

Variable	Younger (n = 12)	Older (n = 12)	*p*-Values
Age (years)	22 ± 2	59 ± 5	*p* < 0.001
Sex	2 males/10 females	4 males/8 females	
Body mass (kg)			Group *p* < 0.001
PRE	68.0 ± 10.3	96.1 ± 13.6	Time *p* = 0.028
POST	69.1 ± 11.2	97.4 ± 15.1	G × T *p* = 0.807
DXA LSTM (kg)			Group *p* = 0.009
PRE	45.0 ± 7.2	54.8 ± 8.9	Time *p* < 0.001
POST	46.6 ± 7.6	56.0 ± 8.9	G × T *p* = 0.454
VL thickness (cm)			Group *p* = 0.007
PRE	2.32 ± 0.32	1.93 ± 0.50	Time *p* = 0.013
POST	2.54 ± 0.33	2.04 ± 0.34	G × T *p* = 0.364
Energy intake (kcal/d)			Group *p* = 0.146
PRE	1409.8 ± 431.5	1763.7 ± 566.6	Time *p* = 0.804
POST	1438.6 ± 369.2	1705.1 ± 597.5	G × T *p* = 0.470
Fat intake (g/d)			Group *p* = 0.337
PRE	64.9 ± 17.7	73.1 ± 24.2	Time *p* = 0.144
POST	58.4 ± 21.6	67.4 ± 26.1	G × T *p* = 0.921
Protein intake (g/d)			Group *p* = 0.010
PRE	54.2 ± 18.6	87.8 ± 31.8	Time *p* = 0.003
POST	75.7 ± 17.4	107.9 ± 46.3	G × T *p* = 0.909
Carbohydrate intake (g/d)			Group *p* = 0.355
PRE	151.9 ± 75.6	189.4 ± 80.1	Time *p* = 0.331
POST	154.6 ± 43.9	168.6 ± 63.9	G × T *p* = 0.213
Fiber intake (g/d)			Group *p* = 0.621
PRE	13.9 ± 6.9	14.2 ± 5.9	Time *p* < 0.001
POST	21.8 ± 5.5	18.5 ± 10.9	G × T *p* = 0.208
Sugar intake (g/d)			Group *p* = 0.098
PRE	54.4 ± 47.2	75.8 ± 44.4	Time *p* = 0.352
POST	43.3 ± 17.9	71.9 ± 37.4	G × T *p* = 0.647

Legend: Data are mean ± standard deviation values, with significant main effects of age group, time, and the group × time (G × T) interaction *p*-values being presented. Note: Age was analyzed using an independent samples *t*-test. Abbreviations: DXA LSTM, lean/soft tissue mass determined using dual-energy X-ray absorptiometry; VL, vastus lateralis.

## Data Availability

Data can be obtained from the corresponding author upon reasonable request.

## Abbreviations

The following abbreviations are used in this manuscript:

ASV	Amplicon Sequence Variant
BMI	Body Mass Index
DXA	Dual-Energy X-ray Absorptiometry
ET	Endurance Training
FABP	Fatty Acid Binding Protein
F/B	Firmicutes to Bacteroidetes
FDR	False Discovery Rate
GC	Gas Chromatography
G × T	Group × Time
HMC	High Microbiome-Accessible Carbohydrate
IRB	Institutional Review Board
LBP	Lipopolysaccharide Binding Protein
LBM	Lean Body Mass
LMC	Low Microbiome-accessible Carbohydrate
LRG1	Leucine-Rich Alpha-2 Glycoprotein 1
LSTM	Lean Soft Tissue Mass
NDSR	Nutrition Data System for Research
OTU	Operational Taxonomic Unit
PCR	Polymerase Chain Reaction
PRE	Pre-intervention
POST	Post-intervention
RPE	Rating of Perceived Exertion
RT	Resistance Training
SCFA	Short-Chain Fatty Acid
USG	Urine Specific Gravity
VL	Vastus Lateralis
